# Editorial: The Golden Future in Medicinal Chemistry: Perspectives and Resources From Old and New Gold-Based Drug Candidates

**DOI:** 10.3389/fchem.2021.665244

**Published:** 2021-03-18

**Authors:** Lara Massai, Sanja Grguric-Sipka, Wukun Liu, Benoît Bertrand, Alessandro Pratesi

**Affiliations:** ^1^ Laboratory of Metals in Medicine (MetMed), Department of Chemistry “U. Schiff”, University of Florence, Florence, Italy; ^2^ Faculty of Chemistry, University of Belgrade, Belgrade, Serbia; ^3^ School of Pharmacy, Nanjing University of Chinese Medicine, Nanjing, China; ^4^ Sorbonne Université, CNRS, Institut Parisien de Chimie Moléculaire (IPCM), Paris, France; ^5^ Department of Chemistry and Industrial Chemistry (DCCI), University of Pisa, Pisa, Italy

**Keywords:** gold complexes, anticancer compounds, mode-of-action, protein metalation, anticancer immunity, mass spectrometry, dihydrofolate reductase, enzyme inhibition

Gold compounds started to play an important role in modern medicine after the discovery of the efficacy of some gold salts against tuberculosis strains by Robert Koch (Sigel et al., 2018). In the 20’s of the last century, Jacques Forestier introduced the idea of using some gold(I) polymeric thiolates for the cure of rheumatoid arthritis (RA), which lead to the birth of gold-based treatments for RA (Kean et al., 1985). However, the turning point had been represented by the development of the orally-administrable Au(I)-based drug auranofin (AF) by Sutton (Sutton, 1986).

Between the end of 1970s and the mid-1980s, the first scientific studies highlighted the *in vitro* antiproliferative properties of auranofin against cancer cells (Mirabelli et al., 1985). This cornerstone finding, also in the light of the heavy side effects of the platinum-based chemotherapy, triggered the interest of the scientific community towards a possible “repurposing” of auranofin as prospective anticancer drug.

In the following years, gold compounds have received large attention, and an impressive number of structurally diverse gold(I) and gold(III) complexes were prepared, such as those stabilized by phosphines or by nitrogen donors, that were found to induce important anticancer effects both *in vitro* and *in vivo* (Marzo et al., 2019). Thanks to several mechanistic studies, preferential gold binding to proteins, instead of DNA, was demonstrated with the occurrence of different pathways to cytotoxicity. Mitochondria and oxidative phosphorylation’s pathways emerged as the primary intracellular targets for cytotoxic gold complexes (Scalcon et al., 2018). Moreover, inhibition of the seleno-enzyme thioredoxin reductase (TrxR) seems to be a common mechanistic trait to explain the antiproliferative actions of several gold complexes, as strong TrxR inhibition may eventually lead to apoptosis (Magherini et al., 2018).

Although the ferment that animates the scientific community on the investigation of gold-based drug candidates, the real and entire picture of the involved mechanisms and modes of action for these compounds remains largely undepicted.

To deepen the knowledge on the gold-based complexes, this Research Topic encloses some fundamental contributions from worldwide outstanding researchers working in this field. In particular, the areas covered range across the synthesis and characterization of new gold N-heterocyclic carbenes (Au-NHCs), to the study of protein metalation processes, to the discovery of new mode-of-action, and up to the search of unexplored molecular targets.

In the furrow of the research on the forefather of gold-based compounds, namely auranofin, Tolbatov et al. described an integrated experimental and theoretical study on the reactivity of AF and its iodide derivative (AF-I) toward relevant amino acid residues or their molecular models, such as the histidine, cysteine, methionine, and selenocysteine, that may represent the possible binding sites. Interestingly, the energetic barriers for the bond formation between the gold compounds and the targets have been calculated through computational methods. The obtained results pointed out that the replacement of the thiosugar moiety with iodide significantly affects the overall reactivity toward the selected targets, contributing to enhance the antitumoral activity of AF-I compared with AF.

A very informative analytical technique such as the high-resolution ESI mass spectrometry, has been successfully applied by Massai et al. to the study of protein metalation by a selected panel of Au(III) complexes. These compounds were reacted with two representative proteins, i.e. human serum albumin and human carbonic anhydrase I, and with the C-terminal dodecapeptide of TrxR. ESI-MS analysis permits to elucidate the nature of the resulting metal–protein adducts from which the main features of the occurring metallodrug–protein reactions can be inferred. In some cases, MS data have been integrated with other independent biophysical measurements for a comprehensive description of the occurring processes.

Because of their oxidizing character, Au(III) complexes are considered an interesting and promising class of potential anticancer compounds involving, beyond the protein metalation, probably also the oxidative cell stress. Radisavljević and co-workers summarized in their review the chemistry of some mononuclear and polynuclear gold(III) complexes. In particular, a special description has been deserved for gold(III) complexes with nitrogen-donor inert ligands and their kinetic behavior toward different biologically relevant nucleophiles. Mechanisms of interaction with DNA, serum albumin, and cytotoxic activity have been thoroughly discussed also in the light of computational calculations.

To date, a great number of different structures of gold compounds have been investigated for their anticancer properties and, among these, Au-NHCs hold great potential. It has been demonstrated that the antiproliferative effects of several gold NHCs were related to their antimitochondrial effects associated with potent inhibition of TrxR. Tong et al. in their review have masterfully summarized the efforts spent in the last years not only on the study of Au(III)-NHC, but also on porphyrin and pincer-type cyclometalated gold complexes. From this description, emerge the anticancer properties of gold(III) compounds and the identification of molecular targets involved in the mechanisms of action. Moreover, particular attention has been devoted to the chemical formulation strategies that have been adopted for the delivery of cytotoxic gold compounds, and for ameliorating the *in vivo* toxicity.

Another class of drug candidates, i.e., imidazolate phosphane gold(I) compounds, has been studied by Galassi and coworkers. They performed enzymatic assays on the inhibition potential of the studied compounds toward the dihydrofolate reductase (DHFR), an enzyme involved in metabolic pathways crucial for cancer cell’s growth and survival and overexpressed in tumor cells. The results have been compared to those obtained for TrxR, the most recognized molecular target for gold compounds.

As completion of this Research Topic’s overview, the role of gold compounds in anticancer immunity has been elucidated in a review by Yue et al., that summarizes the complex relationship between various gold derivatives and the immune system. Gold complexes seem to reverse tumor immune escape and directly facilitate the functions of immune cells, leading to enhanced anticancer effects. In particular, the paper described the antitumor effects of gold drugs and their relationships with various aspects of antitumor immunity, including innate immunity, adaptive immunity, immunogenic cell death, and immune checkpoints, as well as their roles in adverse effects.

This Research Topic aims to give to the readership a new point of view on the current research on gold-based drug candidates, covering different aspects, from the synthesis of new molecules to the study of their relationship with biomolecular targets and the immune system. As Guest Editors, we believe that this collection could be useful to disseminate the last advancements in the research of gold anticancer drugs.
